# Public health communications and alert fatigue

**DOI:** 10.1186/1472-6963-13-295

**Published:** 2013-08-05

**Authors:** Janet G Baseman, Debra Revere, Ian Painter, Mariko Toyoji, Hanne Thiede, Jeffrey Duchin

**Affiliations:** 1 Department of Epidemiology, School of Public Health, University of Washington, Seattle, WA, USA; 2 Department of Health Services, School of Public Health, University of Washington, Seattle, WA, USA; 3 Public Health-Seattle & King County, Seattle, WA, USA

## Abstract

**Background:**

Health care providers play a significant role in large scale health emergency planning, detection, response, recovery and communication with the public. The effectiveness of health care providers in emergency preparedness and response roles depends, in part, on public health agencies communicating information in a way that maximizes the likelihood that the message is delivered, received, deemed credible and, when appropriate, acted on. However, during an emergency, health care providers can become inundated with alerts and advisories through numerous national, state, local and professional communication channels. We conducted an alert fatigue study as a sub-study of a larger randomized controlled trial which aimed to identify the most effective methods of communicating public health messages between public health agencies and providers. We report an analysis of the effects of public health message volume/frequency on recall of specific message content and effect of rate of message communications on health care provider alert fatigue.

**Methods:**

Health care providers enrolled in the larger study (n=528) were randomized to receive public health messages via email, fax, short message service (SMS or cell phone text messaging) or to a control group that did not receive messages. For 12 months, study messages based on real events of public health significance were sent quarterly with follow-up telephone interviews regarding message receipt and topic recall conducted 5–10 days after the message delivery date. During a pandemic when numerous messages are sent, alert fatigue may impact ability to recall whether a specific message has been received due to the “noise” created by the higher number of messages. To determine the impact of “noise” when study messages were sent, we compared health care provider recall of the study message topic to the number of local public health messages sent to health care providers.

**Results:**

We calculated the mean number of messages that each provider received from local public health during the time period around each study message and provider recall of study message content. We found that recall rates were inversely proportional to the mean number of messages received per week: Every increase of one local public health message per week resulted in a statistically significant 41.2% decrease (p < 0.01), 95% CI [0.39, .87] in the odds of recalling the content of the study message.

**Conclusions:**

To our knowledge, this is the first study to document the effects of alert fatigue on health care providers’ recall of information. Our results suggest that information delivered too frequently and/or repetitively through numerous communication channels may have a negative effect on the ability of health care providers to effectively recall emergency information. Keeping health care providers and other first-line responders informed during an emergency is critical. Better coordination between organizations disseminating alerts, advisories and other messages may improve the ability of health care providers to recall public health emergency messages, potentially impacting effective response to public health emergency messages.

## Background

“Information chaos”—various combinations of information overload, information underload, information scatter, information conflict and erroneous information—is an increasingly cited factor affecting the effectiveness, quality and safety of clinical care [1]. By contributing to increased mental workload and decreased situational awareness in the work place, information chaos may also be a factor in alert fatigue; i.e., health care providers (HCPs) missing or ignoring important messages within the volume of information they have been conditioned to perceive as irrelevant [2]. Previous alert fatigue studies have focused on clinical decision support systems (CDS) and computerized provider order entry (CPOE) systems which aim to improve efficiency and quality of care. These studies have investigated how CDS and CPOE systems alert providers to potential adverse medication events and interactions [3,4], the frequency and factors associated with overriding CPOE alerts [2,5], recommended design approaches for mitigating adverse drug events and alert fatigue [6,7], and unintended consequences of these systems in contributing to information and mis-information overload, role confusion, excessive errors and alert fatigue [8]. In addition to the clinical system messages, HCPs receive public health alerts and advisories, clinical guidelines and updates, and training notifications from professional associations, agencies and organizations [9] and through a system of national, state and local public health communication channels [10].

HCPs serve as partners with public health in surveillance and case investigation activities by providing case reporting and clinical management, preventing excess deaths, treating the injured and mitigating suffering [11]. For example, HCPs were the first to recognize early cases of inhalation anthrax in 2001 [12] and first to identify dengue outbreaks in Hawaii, Florida and Texas between 2001–2011 [13]. In an emergency, public health agencies rely on HCPs as both frontline responders and as trusted and preferred communicators of health information to the public during an emergency [14-16].

Ensuring that timely, accurate, trustworthy and context-relevant information can be “heard” by HCPs within their information chaotic environments is essential to an effective public health communication program [17,18]. However, while their intention is to reduce information chaos, the growing variety of public health agency and other messaging systems may be increasing, rather than reducing, communication challenges for HCPs [9]. Poor coordination between agencies can contribute to inconsistent and/or conflicting information as well as redundant messaging between public health agencies and HCPs, as demonstrated by communication issues identified in the 2008 Salmonella Saint Paul outbreak response [19]. Similar issues were recently documented by a process analysis and survey conducted with primary care providers in Utah following the first wave of the 2009 influenza pandemic. A majority of providers stated that: 1) they received an overwhelming amount of email from public health authorities as well as health care organizations and 2) they desired less frequent, more concise and locally relevant emergency communications during a public health emergency [20]. Despite recognition of these problems, little is known about the potential for HCP alert fatigue from public health communications.

The reach (Rapid Emergency Alert Communication in Health) study is a randomized controlled trial to systematically evaluate and compare the effectiveness of mobile (SMS) and traditional (email, fax) communication strategies for sending public health messages to health care providers—physicians, pharmacists, nurse practitioners, physician assistants and veterinarians. The study aims to identify which communication modality is most effective for dissemination of health alerts and advisories between public health agencies and HCPs in order to improve emergency preparedness and response. The findings reported in this paper are a reach sub-study involving one study site. The objective of the analysis described in this paper is to measure the effects of public health message volume on recall of specific message content as a measure of HCP alert fatigue.

## Methods

### Ethics

Study protocols were approved by the UW Institutional Review Board (Minimal Risk Category 7).

### Design and setting

The reach study is a multi-site randomized control trial conducted by the University of Washington (UW) across three sites in partnership with local (King County, WA and Spokane County, WA) or state (State of Montana) public health agencies. The findings reported in this paper are a reach sub-study involving the King County, WA study site. Between October 2009 and January 2011, the reach study was conducted in King County, WA, the most populous county in Washington State, and the 14th most populous in the United States [21]. HCPs were recruited and randomized to receive public health messages via email, fax, short message service (SMS delivered to a cell phone) or to a control group that did not receive messages. HCPs were blindly randomized regardless of ability to receive the group intervention; for example, HCPs who did not provide a fax number at enrollment could be blindly randomized into the fax group. Investigators, data analysts and interviewers were blinded to randomization groups.

### Eligibility

Providers were eligible for the study if they were an active, practicing physician in a primary care related specialty, nurse practitioner, pharmacist, physician assistant or veterinarian in King County, WA. Providers were excluded if they were inactive or retired, involved with the study or worked for the site’s public health agency. Physicians were oversampled due to historically low study response rates from this population. Of 7,281 eligible HCPs in King County, 530 enrolled in the study and 528 were included in this analysis. Table 1 provides HCP demographics.

**Table 1:** Demographic characteristics of study participants in King County

	**N**	**%**		**Mean**	**SD**		**Male/Total**	**% Male**
							**Total**	
**Provider**			**Age**			**Gender**		
**type**								
ARNP	126	23.86		48.6	10.5		12/121	9.9
MD	209	39.58		48.8	9.5		106/200	53.0
PA	35	6.63		46.0	11.0		10/33	30.3
PHRM	103	19.51		45.9	12.4		39/93	41.9
VET	55	10.42		44.7	11.0		16/53	30.2
**Missing**	0			64			28	

Provider Type: *ARNP* Advanced Registered Nurse Practitioner, *MD* Physician, *PA* Physician Assistant, *PHRM* Pharmacist, *VET* Veterinarian.

### Intervention

**Figure 1:**
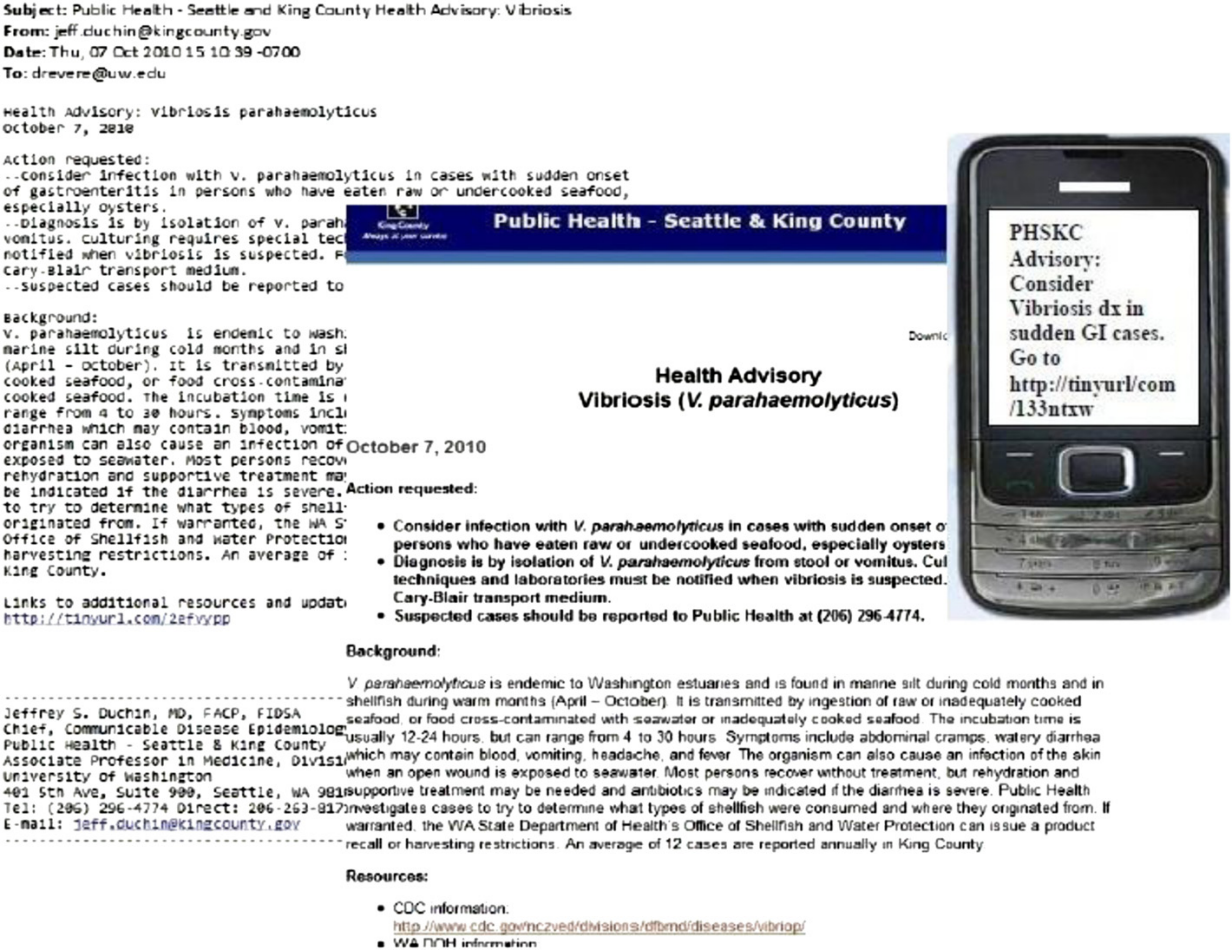
**Example reach study message: Full message (left) sent via email and fax and truncated messages (right) sent via SMS.** All messages contained a link to a web page (center).

Between March 2010 and January 2011, messages based on real events of public health significance were sent quarterly to enrolled HCPs. All messages included a link to a public health web page with additional information. Message format varied by delivery mechanism as seen in Figure 1 Email and fax messages included standard content while those delivered via SMS were limited to 160 characters.

### Intervention assessment

**Figure 2:**
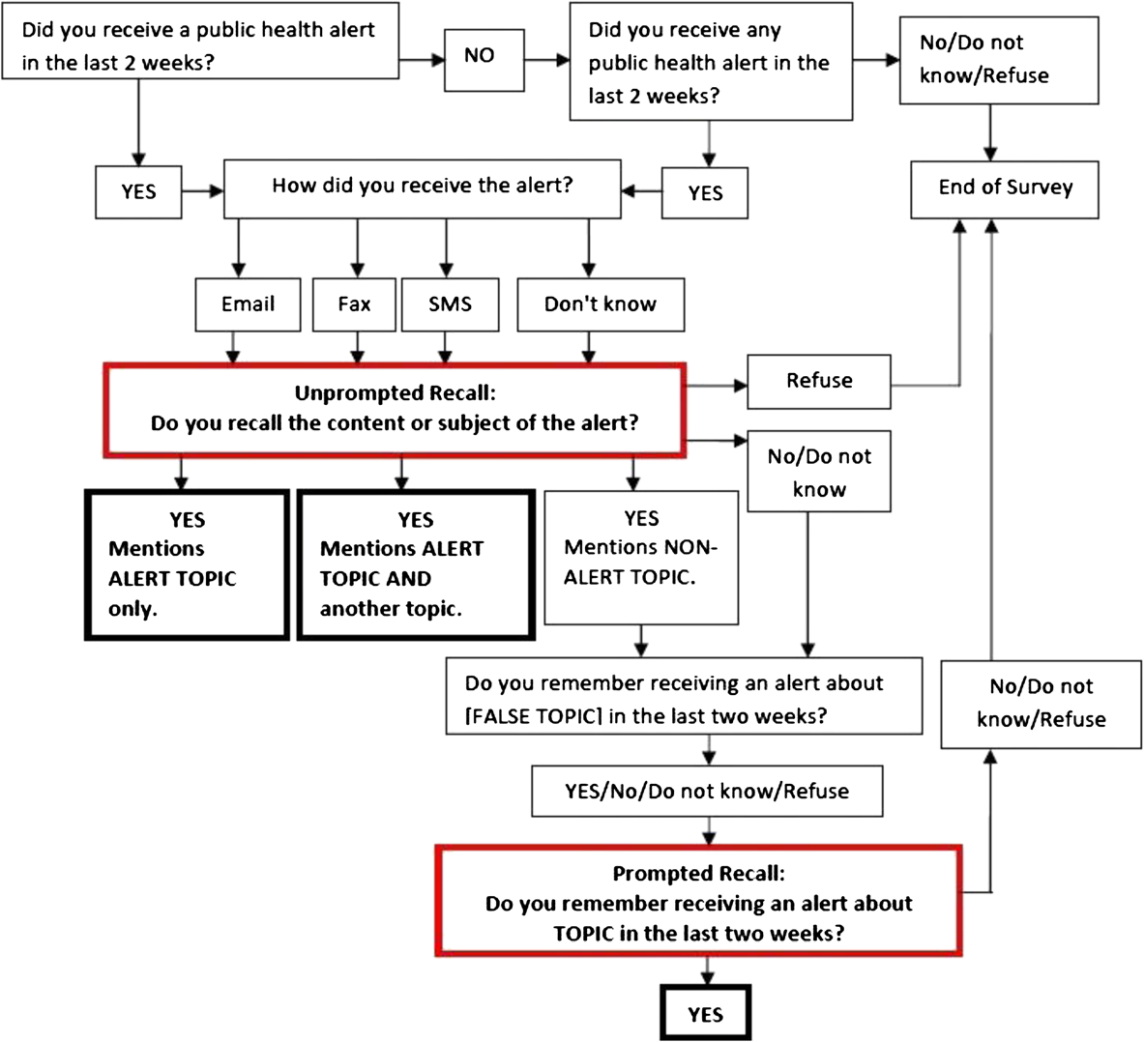
Phone interview questions used to measure outcome measures of message recall.

Follow-up phone interviews were conducted 5–10 days after message delivery date. Interviews consisted of approximately six questions that elicited information about message receipt, recall of its content and perceived credibility and trustworthiness of the message and its source. Figure 2 illustrates the interview protocol. The interview included two methods for determining whether HCPs who reported receiving the message recalled its topic correctly (boxes outlined in red on Figure 2): unprompted recall and prompted recall. *Unprompted recall* measured whether the HCP correctly identified the reach message either alone or in combination with another message topic. If HCPs recalled receiving a message but identified a non-reach topic, interviewers utilized the *prompted recall* script. In that script, HCPs were asked first if they could recall receiving a message about a topic which was not related to the content of any messages sent by the reach study or the public health agency (i.e., a “fake” or distractor topic) and then if they remembered the study message topic. HCPs who did not remember receiving a public health message in the previous two weeks, gave an open-ended response that did not match the study message topic or claimed to remember the “fake” topic were classified as not correctly recalling the message. HCPs that correctly remembered the reach study message topic with and without prompting were classified as having correctly recalled the message.

### “Noise” data collected

**Figure 3:**
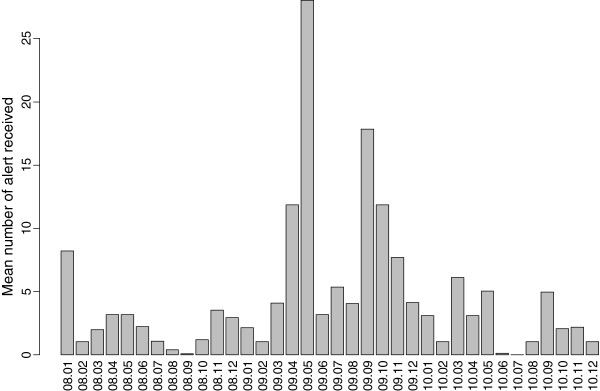
**Number of messages sent over public health agency email listserv per month from 2008–2010.** The two waves of the 2009 H1N1 pandemic are marked by dramatic increases in messages.

To calculate “noise” during the timeframe of each reach message, HCPs who subscribed to the public health department’s email listservs were identified and the number and topic of messages disseminated by the public health department’s listserv 4 weeks prior to and 3 weeks after each study message were documented. Of the sample total of 528, 23.7% of HCPs were identified as subscribers to at least one public health listserv (see Table 2). Public health department messages included a range of alerts, advisories, guidelines and subscription information. Figure 3 illustrates the variation in number of public health messages sent per month prior to and during the study period.

**Table 2:** Listserv subscription of King County health care providers

**Listserv subscription?**	**N**	**%**
Yes	125	23.67
No	403	76.33
Missing	0	
	**Mean**	**SD**
Messages received per week from listserv*	0.21	0.54
Missing	0	

* For periods prior to communication of study messages.

### Data analysis approach

All analyses were conducted using R v2.10.0 [22]. Basic HCP demographic data were compiled based on HCP information provided at enrollment and interview questions regarding age and gender (Table 1). Proportions of HCPs recalling receipt of study messages and correctly recalling study message topics with and without prompting were calculated using responses from follow-up interviews.

### Data analysis methods

To examine the main effect of message rate on alert fatigue, we analyzed the effects of message rate on the probability of correctly recalling content of study messages among those who remembered receiving messages. A HCP was classified as having correctly recalled the reach study message topic if the HCP: 1) Recalled receiving a message without prompting and the topic correctly matched the reach study message topic; or 2) Did not recall receiving the “fake” topic but did recall the reach study message topic. To discount the effects of “agreeability bias”, HCPs who recalled receiving a message on the “fake” topic and required prompting for the study message topic were not counted as correctly recalling receipt of the study message. All other responses were classified as not correctly recalling the message.

Analysis was conducted using generalized linear models (GLM) with Binomial outcomes and the logit link function with exchangeable autocorrelations within providers using generalized estimating equations (GEE) in using the function ‘gee’ in the ‘gee’ package. Robust standard errors were used to calculate z scores and p-values. Data from the first message were not included in this analysis because questions prompting HCP recall with the study message topic were only used on interviews for messages 2–5. Message number was included in the model to control for differences in recall or “memorability” of the study message topic.

Exploratory data analysis of the data indicated that one of the (blinded) randomization groups was obviously the control group due to a large failure rate in recall. For the alert fatigue analysis and results that follow, the control group was excluded since the alert fatigue analysis focused on an HCP’s ability to recall whether our message was received amidst the “noise” of other messages they received and there was no mechanism by which message rate could influence recall of the study messages. Two covariates other than message rate— message number and (blinded) randomization group—were included in the main analysis. Our analysis investigated: 1) HCP rates of message recall for both prompted and unprompted responses; 2) estimated effect of increases in message rate on prompted recall; and 3) differences in the effect of message rate on recall between provider types, gender, age and randomization group (message delivery method).

## Results

### Message recall

We found substantial variation in the rates at which HCPs recalled the study message topic for both prompted and unprompted responses (see Table 3 for rates of recall for each message). Recall rates were inversely proportional to the mean number of messages received per week. A substantial proportion (28.1%) of HCPs who recalled the study message topic after prompting also recalled the “fake” topic; this did not vary substantially across messages.

**Table 3:** Rates of message recall by message and outcome type

**Outcome**	**Message**	**Message**	**Message**	**Message**
	**2**	**3**	**4**	**5**
Mean number of messages received per week by participants belonging to any listserv.	1.13	0.33	0.35	0.68
Recall content of study message (coded open ended question) among those that could recall content	66/171	139/181	106/191	118/212
	66/171	139/181	106/191	118/212
“Fake”/Distractor topic recalled when study topic was recalled	32.2	27.4	26.1	29.3
	19/59	20/73	24/92	17/58
Correct recall of study message by prompting or by open ended response among participants who recall receiving any message	52.7	74.9	62.5	57.3
	87/165	176/235	150/240	134/234
Correct recall of study message by prompting or by open ended response, over all participants interviewed	24.4	42.4	36.6	35.6
	87/356	176/415	150/410	134/376

### Effect of message rate on alert fatigue

Analysis of prompted recall (Table 4) was calculated using a generalized estimating equation estimation of a generalized linear model (binomial family with logistic link function) with prompted recall as the outcome and weekly message rate and message number as covariates. We found that every increase of one message per week resulted in a statistically significant 41.2% decrease in the odds of recalling the content of the study message (p < 0.01), 95% CI [0.39, .87]. Within HCP correlation was small at 0.10. Differences in the effect of message rate on recall between provider types, provider gender and provider age were examined using analysis of covariance of generalized linear models by separately including each covariate as an interaction term with message rate. We detected no effect of message rate on recall between provider types, gender or age.

**Table 4:** Analysis of prompted recall

**Covariates**	**Effect**	**Coefficient**	**Robust**	**Robust**
	**(odds scale)**		**standard error**	**z-score**
(Intercept)		0.755	0.227	3.332
weekly message rate	0.588	−0.531	0.209	−2.545
Message 3	1.853	0.617	0.261	2.361
Message 4	0.999	−0.001	0.265	−0.005
Message 5	1.037	0.036	0.244	0.147
Within participant correlation: 0.10				

The inclusion of the additional interaction term reduced the residual deviance by 0.38, 2.80 and 0.08 respectively on 4, 1 and 1 degrees of freedom; these reductions are all small in magnitude and none are statistically significant. An analysis of randomization group on alert fatigue demonstrated no effect of communication channel on alert fatigue.

## Discussion

To our knowledge, this is the first study to document the effects of alert fatigue on HCPs’ recall of information. The number of local public health messages significantly decreased the odds of HCPs correctly remembering the receipt and content of a study message, and suggests that alert fatigue and information overload may inhibit the ability of HCPs to respond effectively to messages during a public health emergency when volume of messages may be higher. The higher than expected recall of a “fake” topic, despite its irrelevance to any recent public health message, may indicate a degree of HCP role confusion or uncertainty around public health messages or it could be an outcome related to information chaos in the health care setting.

Information delivered too frequently and/or repetitively through multiple communication channels also may negatively affect the ability of HCPs to recall public health messages. It should be noted that six months prior to the date of this study, King County HCPs on local public health listservs were exposed to high volumes of health alerts and advisories during the initial onset of the 2009 H1N1 pandemic. This “noise” may have desensitized HCPs to information delivered through the public health emergency message system. We also did find a general trend of improvement in recall of message receipt and recall of content over time, which could be attributed to reduced message volume in the time period before the study message and could indicate that HCPs recovered from the high volume of messages sent during the H1N1 pandemic.

It might seem logical that an electronic health record (EHR) that provides contextually relevant information to clinicians would also provide a delivery mechanism for population-based information. However, it is far from clear that this information would not become part of the clinical “noise” and contribute to increased information chaos in the clinical environment. Alert fatigue studies conducted in clinical environments have documented that over 90% of medication safety/drug-drug interaction alerts are overridden in electronic decision support systems without a physician even looking at the alert [23]; pharmacists have also been found to override one-third of life-threatening drug-drug interactions [24]. Low alert specificity, unclear information content, perceived lack of relevance, information overload and unnecessary disruptions to workflow are cited as reasons for ignoring alerts [2]. While the opportunity to leverage EHRs to disseminate public health alerts and advisories is promising, more research is needed to identify the most effective means of delivering these messages to HCPs, as well as how to communicate with HCPs who do not work in an environment with an EHR.

Also more research is needed to identify public health message sources of greatest value to HCPs. Anecdotal evidence from open-ended interview responses suggested that at least some HCPs received messages from other sources, such as the CDC, FDA, FEMA, as well as professional organizations such as the Washington State Medical Association and internal employer communications during the study period. We were unable to accurately document these additional message sources, although source of message may impact the likelihood of message recall.

### Limitations

Our study has several limitations.

Study messages, while timely, were not emergency alerts and, thus, may have been perceived as less relevant, important or “memorable” than other public health agency messages HCPs received during the same time frame. In addition, we varied the day of the week that study messages were sent. It is possible that messages sent on certain days are better recalled by HCPs and that our messages were sent on lower recall days. To study this phenomenon would require communication of multiple study messages on each day of the week—a wholly different study than we conducted—thus we do not have data to determine whether HCPs experience lower or higher recall days.

The primary explanatory variable of interest, message rate, is an uncontrolled variable. Our ability to estimate the effect of message rate depends on our ability to separate out the effects of alert fatigue from the “memorability” effects of individual messages. This depends on the variability in the number of messages received between participants, and a large part of this variability comes from differences based on public health listserv membership. To the extent that systematic differences exist between providers who do and do not belong to public health listservs, biases may exist in our estimates. We identified HCPs who subscribed to public health listservs by comparing listserv and study contact information. It is possible that our listserv identification was not accurate as HCP contact information may have changed or HCPs may have subscribed to a listserv after the study began, which in turn means our estimates of the rate at which messages were received by participants may have been underestimated. The effect of such bias would be to underestimate the effect size of message rate on message recall.

We also are unable to document whether reach subjects actually received the local public health listserv communications or whether a listserv message was shared with other HCPs; we can only document that a public health listserv message was sent to the HCP. This highlights an issue for public health messaging through lists—whether email, fax or SMS—in general. Most public health listservs require HCPs to opt in to receiving messages and the burden of maintaining correct contact information rests on public health agency staff. A clinic, hospital or other health care organization that maintains a directory of provider contact information may be more a more efficient mechanism for disseminating public health messages and communicating emergency information to its HCPs. However, this raises the question of whether a public health message has the authority or novelty to be “heard” within the usual clinical noise and would need further investigation.

While this study contributes to the evidence base for improving public health agency emergency communications with HCPs, we cannot correlate failure to recall message topic with inability to act on the information received, such as HCPs communicating information further downstream by, for example, sharing email with colleagues or disseminating information to patients or the public. Recent disasters have highlighted effective communication as essential in public health agencies’ ability to protect populations during emergencies; these events can increase information chaos in the health care environment. More research is needed to understand the impacts of the method of message delivery, the source of the message, the type of information being disseminated and the health care organizational context to guide or improve the practice of communication between public health agencies and HCPs before, during and after a public health emergency.

## Conclusions

Keeping health care providers and other first-line responders informed during an emergency is critical. Implementing measures to avoid redundant and conflicting messages from multiple agencies may help reduce alert fatigue. Delivering public health alerts and advisories too frequently and/or repetitively may have a negative effect on the ability of HCPs to effectively recall and, potentially, to respond to public health emergency messages. Our results suggest that public health agencies should consider the potential impacts of timeliness of information and frequency of communications in emergency planning to reduce the potential for alert fatigue and information chaos during public health emergencies.

## Competing interests

The authors declare that they have no competing interests.

## Authors’ contributions

JB, DR, IP, JD and HT conceived of the study. JB, DR, IP and HT equally participated in identification and definition of outcome measures. IP led the data analysis with participation by JB and DR. JB, DR and MT authored the overall manuscript with contributions by IP, HT and JD. All authors read and approved the final manuscript.

## Pre-publication history

The pre-publication history for this paper can be accessed here:

http://www.biomedcentral.com/1472-6963/13/295/prepub
